# Development, Validation, and Application of a Novel Method for the Analysis of Vitamin E Acetate and Other Tocopherols in Aerosol Emissions of E-Cigarettes, or Vaping Products Associated With Lung Injury

**DOI:** 10.3389/fchem.2021.730954

**Published:** 2021-08-05

**Authors:** Andrew Puetz, Maria Morel Espinosa, Clifford Watson, Benjamin C. Blount, Liza Valentín-Blasini

**Affiliations:** Tobacco and Volatiles Branch, Division of Laboratory Sciences, National Center for Environmental Health, U.S. Centers for Disease Control and Prevention, Atlanta, GA, United States

**Keywords:** vitamin E acetate, tocopherols, EVP aerosol, LC-MS/MS, EVALI

## Abstract

E-cigarette, or vaping, product (EVP) use has increased dramatically in the United States over the last 4 years, particularly in youth and young adults. Little information is available on the chemical contents of these products. Typically, EVPs contain an active ingredient such as nicotine, CBD, or THC dissolved in a suitable solvent that facilitates aerosol generation. One EVP solvent, vitamin E acetate (VEA), has been measured in EVP liquids associated with lung injury. However, no validated analytical methods for measuring VEA in the aerosol from these devices was previously available. Therefore, we developed a high throughput isotope dilution LC-MS/MS method to simultaneously measure VEA and three other related tocopherols in aerosolized EVP samples. The assay was precise, with VEA repeatability ranging from 4.0 to 8.3% and intermediate precision ranging from 2.5 to 6.7%. Similar precision was obtained for the three other tocopherols measured. The LODs for the four analytes ranged from 8.85 × 10^−6^ to 2.28 × 10^−5^ μg analyte per mL of aerosol puff volume, and calibration curves were linear (*R* ^2^ > 0.99). This method was used to analyze aerosol emissions of 147 EVPs associated with EVALI case patients. We detected VEA in 46% of the case-associated EVPs with a range of 1.87 × 10^−4^–74.1 µg per mL of aerosol puff volume and mean of 25.1 µg per mL of aerosol puff volume. Macro-levels of VEA (>0.1% w/w total aerosol particulate matter) were not detected in nicotine or cannabidiol (CBD) products; conversely 71% of the EVALI associated tetrahydrocannabinol (THC) products contained macro-levels of VEA. Trace levels of other tocopherol isoforms were detected at lower rates and concentrations (α-tocopherol: 41% detected, mean 0.095 µg analyte per mL of aerosol puff volume; γ-tocopherol: 5% detected, mean 0.0193 µg analyte per mL of aerosol puff volume; δ-tocopherol: not detected). Our results indicate that VEA can be efficiently transferred to aerosol by EVALI-associated EVPs vaped using a standardized protocol.

## Introduction

In recent years the use of e-cigarettes, or vaping products (EVPs) have dramatically increased^1^. More than eight million U.S. adults reported using these products on a regular basis^1^ (Creamer MR, 2019). The use of nicotine containing EVPs could potentially benefit adult smokers if used as a complete substitute for traditional cigarettes and other combusted tobacco products rather than dual use (Shahab et al., 2017). However, EVP use increases disease risk for those who are not already using tobacco products (Orzabal and Ramadoss, 2019; Wang et al., 2020). Research is needed to better understand potential long-term health effects of inhaling EVP aerosols, including solvents, additives, and diluents (Ghosh and Drummond, 2017; Bhatnagar et al., 2019; Hajek et al., 2019). One of the main challenges for assessing the potential health impacts of EVP use is the accessibility to e-liquid components and formulations through “informal” and individualized marketing (Zhu et al., 2014) Decriminalization of cannabinoids for medical and non-medical purposes by some states facilitated a surge in use of EVPs for vaping cannabis as these products are more available in some markets^2^ (Pacula and Smart, 2017; Mcnamara, 2020).

From August 2019 to February 2020 an outbreak of e-cigarette, or vaping, product use associated lung injury (EVALI) occurred across the United States: at least 2,807 people were hospitalized, and 68 deaths reported in 29 states. Most EVALI cases were <35 years of age and previously healthy (Lozier et al., 2019; Moritz et al., 2019). Most patients that were hospitalized reported using e-liquid products containing THC (Butt et al., 2019; Lozier et al., 2019; Siegel et al., 2019). These lung injury cases have been associated to the use of vitamin E acetate (VEA)-containing EVPs (Duffy et al., 2020). Analysis of bronchoalveolar lavage fluid from EVALI patients identified VEA accumulation and thus implicated inhaled VEA as the likely cause of the 2019 EVALI lung injury outbreak (Blount et al., 2019; Blount et al., 2020). Multiple trade websites report the use of VEA and medium- chain triglycerides in THC products to enhance quality, appearance, and aroma, as well as a way to lower production cost (Downs, 2019a; Downs, 2019b; Zachary Eisenberg, 2019).

VEA is the shelf-stable synthetic form of vitamin E often used in skin care products and dietary supplements. Oral and topical administration of VEA has been used for years without significant adverse health effects. Because of the 2019 EVALI outbreak, the effects of inhaled VEA are starting to be evaluated. In fact, two recent studies find that mice exposed to VEA emissions develop lung injury and other pathologies similar to EVALI patients (Bhat et al., 2020; Matsumoto et al., 2020)**.** Traditional analysis of VEA and other tocopherols are mainly performed through HPLC-UV for cosmetics and foods products intended for dermal application or ingestion (Cunha et al., 2006; Bustamante-Rangel et al., 2007; Almeida et al., 2009; Nada et al., 2010; Şeker et al., 2012; Cortés-Herrera et al., 2019; Sadrykia et al., 2019). Recently, the analysis of VEA in EVP liquids was reported using screening and targeted GC-MS and LC-MS/MS assays^3^ (Health, 2019). No methods existed for the analysis of VEA and other tocopherols in aerosol emissions of e-liquid products samples, thus here we report on the development and validation of an analytical method for VEA and other tocopherols in aerosol emissions samples. The efficacy of the method is subsequently demonstrated by analysis of aerosol emissions from EVPs associated with EVALI case patients.

## Experimental

### Materials and Methods

Methanol (CAS# 67-56-1; LC-MS/MS grade) purchased from Fischer Scientific was mixed with deionized water from an ultrapure water purifications system (Aqua Solutions model RODI-C-11BL, Jasper, GA, United States) and formic acid (CAS# 64-18-6; chemical purity: ≥ 98%; ACS reagent) to form a 90% methanol: 10% water and 0.1% formic acid mobile phase.

VEA, DL-alpha Tocopherol acetate (CAS# 7695-91-2, ≥99% purity), (+)-alpha Tocopherol (CAS# 59-02-9, ≥99% purity), (+)-gamma Tocopherol (CAS# 54-28-4, ≥96% purity), delta-Tocopherol (CAS# 119-13-1, ≥99% purity), alpha-Tocopherol-(phenyl-^13^C_6_) (≥99% atom purity, ≥96% compound purity), labeled vitamin E acetate - (trimethyl-d_9_) (≥ 98 atom %; chemical purity: ≥98%) were all purchased from Sigma Aldrich (St. Louis, Missouri, United States).

Stock solutions and calibrators of unlabeled VEA and other tocopherols were prepared by individually weighing neat compounds using a calibrated analytical balance and dissolving each in methanol. Multianalyte working solutions were prepared from individual stocks and stored at −20°C until use. The internal standard stock solutions were prepared in a similar manner using labeled alpha-tocopherol-(phenyl-^13^C_6_) and labeled VEA-(trimethyl-d_9_) in methanol. A working solution containing both labeled tocopherols was prepared in methanol and stored at −20°C.

### Sample Preparation

Aerosol emissions were generated by vaping the EVP liquid samples on a Cerulean CETI-8 e-cigarette vaping machine equipped with button activation switches (Cerulean, Richmond, VA). The vaping machine puff volume was calibrated and verified using a soap bubble flow meter prior to use. Vaping conditions were adopted from the CORESTA Recommended Method No. 81 (CORESTA, Recommended Method Number 81: Routine analytical machine for e-cigarette aerosol generation and collection-definition and standard conditions). Freshly charged batteries were used in an EVP to vape each provided cartridge or liquid. Pre-filled cartridges provided without an EVP were vaped using a Honeystick (Fort Lauderdale, FL, United States) 510 Twist Vape Pen with the battery set at the highest voltage (4 V). When case-associated products included a compatible battery, the provided battery was charged and used at the highest voltage setting for vaping that case-associated cartridge. The aerosol from 15 consecutive puffs from vaped EVPs liquids was trapped on individual pre-conditioned Cambridge filter pads (CFPs; 44 mm) that were purchased from Thermo Fisher Scientific (Waltham, MA, United States) and housed in filter pad holders from Cerulean (Molins PLC, MK, United Kingdom). Custom-made connectors (“mouth pieces”) were fabricated in-house *via* 3D printing technology as needed for non-circular mouth piece geometries. The total particulate matter (TPM) was gravimetrically determined by mass difference of pre- and post-vaping CFP for each sample. EVPs that produced less than 6.5 mg TPM/15 puffs were considered unacceptably low, flagged as a QC failure, and not reportable. Post-vaped CFPs were individually placed into 16 ml amber vials for extraction. CFPs were extracted with 10 ml of methanol on an orbital shaker for 10 min at 160 rpm. Sample extracts were diluted 100-fold prior to tocopherol analysis. Prior to analysis, 100 µL of the dilute solution was spiked with labeled internal standard and diluted with methanol to 1 ml in an autosampler vial.

### Instrumentation

A high-performance liquid chromatography (HPLC) system (Agilent Technologies, Santa Clara, CA, United States) coupled with electrospray tandem mass spectrometry (SCIEX 5500 Triple Quad Applied Biosystems, Foster City, CA, United States) was used to quantitatively measure vitamin E acetate and other tocopherols in trapped aerosol emissions of e-liquids. Chromatographic separation was achieved using isocratic elution at a flow rate of 0.75 ml/min on an XTerra MS C18 column 3.5 µm × 50 mm × 150 mm (Waters Corporation Milford, MA United States) with methanol, water, and formic acid (89.9:10:0.1) as the mobile phase. The eluent from the column was ionized using an electrospray interface to generate and transmit positive ions into the mass spectrometer for selective, quantitative analysis. Analyst software version 1.6.2 (Applied Biosystems, Foster City, CA, United States) was used to operate the HPLC and the 5500 Triple Quad. The mass spectrometer was operated in multiple reaction monitoring (MRM) mode for positive ions. The ion source temperature was set at 350°C and the electrospray ion voltage at 5,500 V. Table 1 presents the optimized MRM transitions used for quantification, confirmation, and internal standard.

**TABLE 1:** MRM transitions and parameters for VEA and tocopherols at a dwell time of 250 ms for all analytes.

Analyte	Transition type	Ion transition	DP (V)	CE (V)	CXP (V)
VEA	Quantitation	473.1→207.1	206	25	16
	Confirmation	473.1→165.1	206	51	12
α-tocopherol	Quantitation	431.2→165.1	61	33	12
	Confirmation	431.2→137.0	61	57	10
δ-tocopherol	Quantitation	403.2→137.0	46	35	10
	Confirmation	403.2→81.0	46	65	10
γ - Tocopherol	Quantitation	417.2→151.1	76	27	12
	Confirmation	417.2→123.0	76	55	14
α-tocopherol-(phenyl-^13^C_6_)	Internal standard	437.2→171.1	56	27	14
VEA-(trimethyl-d_9_)	Internal standard	482.3→216.1	21	25	14

### Quantification

Analyst software 1.6.2 was used for peak integration, calibration, and quantification. Analyte quantification was achieved using the ratio of relative peak area of the analyte to that of the labeled internal standard. Aerosol emissions samples results (instrument output in ng/mL) were normalized by aerosol puff volume to determine analyte yields per puff following the equation below.

$$\text{µg per mL aerosol puff volume}=\;tocopherol_{measured}\;\left(\frac{ng}{mL}\right)x\;\frac{10\;mL}{1000\;\frac{ng}{µg}\;x\;15\;puff\;x\;55\frac{mL}{puff}}$$

where tocopherol measured is the instrument calculated analyte concentration in ng/mL multiplied by the result of the division of the total sample extraction volume of 10 ml by the total puff aerosol volume defined as 15 puffs/pad × 55 ml/puff and a factor of 1,000 to convert ng to µg.

### Quality Control Samples

EVPs are chemically diverse; therefore, we created a diverse surrogate matrix for preparing calibration curves, QC pools and blanks. The surrogate matrix was created by combining the aerosol extract from four different vape liquids, a commercial product (VUSE Solo Menthol), and three custom mixtures (25% squalene/25% squalane/50% mineral oil, 100% CDB oil, and 25% vitamin E acetate/25% medium chain triglycerides/50% hemp oils. The surrogate matrix was vaped using a Vaporin Presidential device and the aerosol trapped using the same technique as for unknown samples. Each pad was extracted with methanol for 10 min in an orbital shaker and combined to produce an 80 ml mixture. The vaped surrogate matrix extract was stored at −20°C. QC samples were prepared daily by individually spiking diluted vaped surrogate matrix extract with known amounts of mixed VEA and tocopherols. Two replicates of a low (QCL) and a high (QCH) level were analyzed per analytical batch. Characterization of each QC level was performed using 20 independent analyses to establish control limits. This QC characterization was subsequently used to evaluate assay performance for each analytical batch based on modified Westgard Rules as described by Caudill et al. (2008). If an analyte failed QC, then none of the results for that analyte in that analysis batch was reportable.

### Calibration

Each analytical batch consisted of a set of seven calibration standards prepared in vaped surrogate matrix extract. The calibration was fit to a weighted 1/× least square model for all analytes generating linear curves with *r* ^2^ > 0.9988. The limit of detection was defined as three times the standard deviation at zero concentration derived from the analysis of six replicates of the three lowest calibration standards (Taylor, 1987). Data are only reported that fall within the calibrated range. Samples exceeding the highest calibration point are diluted and reanalyzed.

### Accuracy, Dynamic Range, Linearity, and Precision

Method accuracy was assessed by spiking the vaped surrogate matrix extract at three different levels of VEA and tocopherols. Six replicates of each level, 200, 400, and 600 ng/ml were used to calculate the analyte recovery. The dynamic range selected covers two orders of magnitude (10–1,000 ng/ml equivalent to 1.21 × 10^−4^–0.0121 µg per mL aerosol emission) to expand the screening capabilities of the assay. Linearity of the dynamic range was evaluated by residual analysis of seven independent curves. Method precision was evaluated as repeatability and intermediate precision of 20 independent QC samples results.

### Method Application

EVP liquid samples were transferred to CDC by FDA and various state health departments for aerosols analysis. Samples that did not contain adequate liquid volume for the assay were not analyzed and the contents saved for liquid analyses. Strict chain of custody was maintained throughout the duration of the study. We applied the validated method to analyze aerosol emissions from 147 EVPs associated with the 2019 U.S. EVALI outbreak. EVP liquid samples were transferred to CDC by FDA and various state health departments for aerosols analysis. Strict chain of custody was maintained throughout the duration of the study. Of those 147 samples, a subset of 138 had reportable corresponding nicotine, CBD, and THC levels. These products were categorized as tetrahydrocannabinol (THC) products if THC ≥0.3% (w/w), nicotine products if nicotine >0.2% (w/w), and cannabidiol (CBD) products if CBD >1% (w/w) and THC <0.3%.

## Results and Discussion

### Method Validation

**FIGURE 1:**
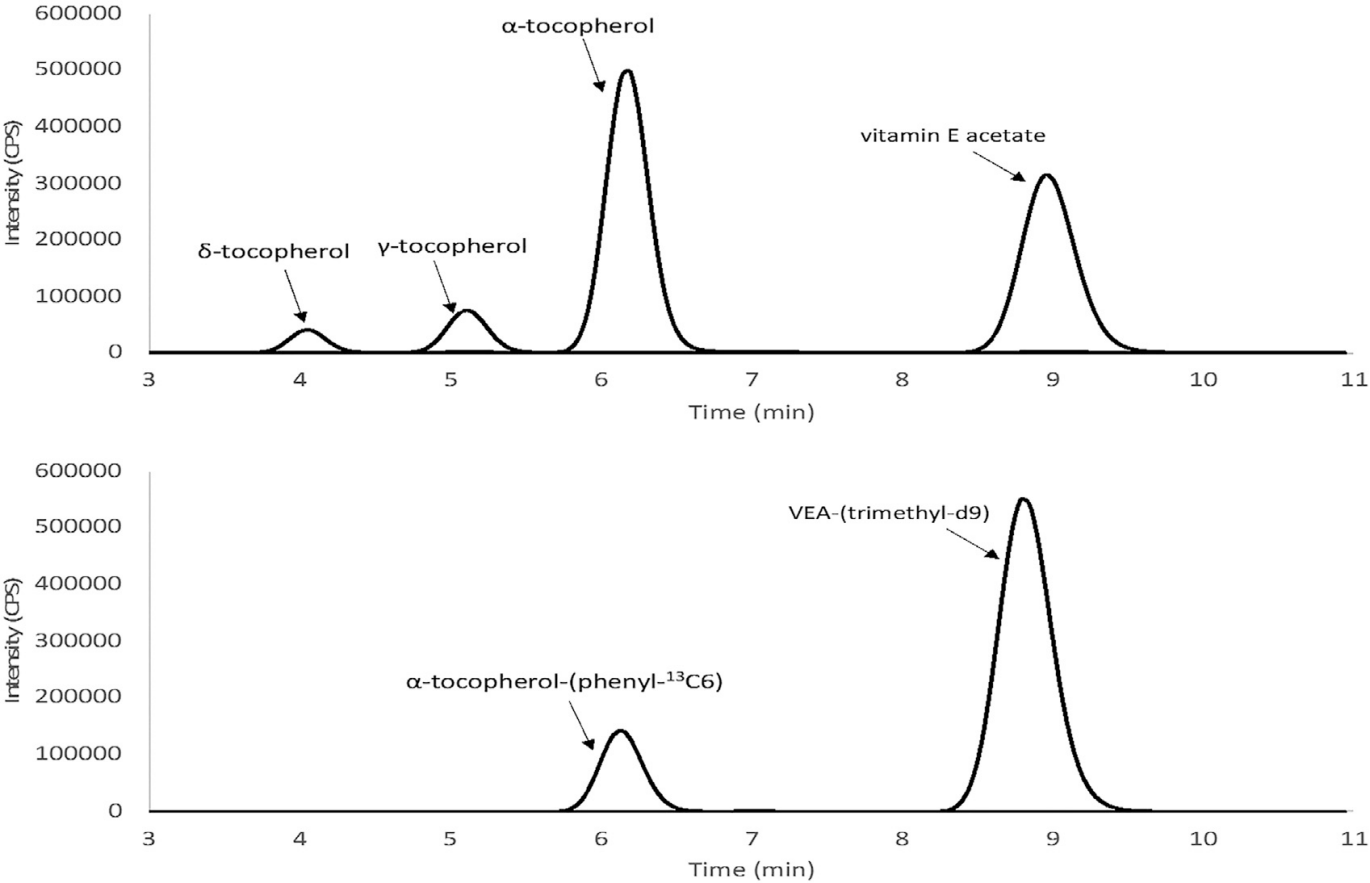
Representative chromatograms of vitamin E acetate (VEA) and other tocopherols spiked into emissions of simulated EVP liquid: **(Top)** VEA and other tocopherols combined quantitation transitions (500 ng/ml) **(bottom)** labeled α-tocopherol (200 ng/ml) and deuterated VEA (200 ng/ml).

We developed a sensitive and quantitative method using LC-MSMS to detect VEA and other tocopherols in aerosol emissions of EVALI case-associated EVPs. Complete chromatographic separation was achieved for the tocopherols without any presence of potential matrix interferences as shown in Figure 1. Method specificity was attained by using isotopically labeled tocopherols to establish the presence of unlabeled tocopherols using both the LC retention time and MS/MS mass selection of the triple quad platform.

Method accuracy was analyzed based on six replicates each of un-spiked and spiked vaped surrogate matrix extract at three different levels of VEA and other tocopherols. Spike recoveries (comparison of spiked calculated result to target concentration) and coefficients of variation (CV) were calculated for each spike level. The mean recoveries for VEA and other tocopherols ranged from 100 to 115% with overall CVs of 4–11% as shown in Table 2. We also evaluated matrix effects by analyzing calibration standards in both methanol and vaped surrogate matrix extract. The average slope of six independent calibration curves in solvent and vaped surrogate matrix extract showed a difference of less than 5% for all analytes. These measures document that the method accurately measures VEA and other tocopherols in aerosol emissions of simulated EVP liquids.

**TABLE 2:** Analyte recovery in EVP vaped surrogate matrix extract at three spike levels.

Analyte	Spike concentration (ng/ml)	Spike recovery (%)	CV, %	Mean recovery (%)	Overall CV, %
VEA	200	102	4.11	100	4.30
	400	98	3.92		
	600	100	4.86		
α-tocopherol	200	111	10.8	107	7.33
	400	109	3.66		
	600	102	7.55		
δ-tocopherol	200	116	11.2	115	11.2
	400	113	11.9		
	600	116	10.5		
γ- tocopherol	200	120	13.3	115	9.88
	400	110	6.82		
	600	114	9.49		

(based on six replicates for each spike level).

Method precision was evaluated as repeatability and intermediate precision from the analysis of 20 independent results for each of two QC levels, QC low (150 ng/ml) and QC high (800 ng/ml), over 10 days (Table 3). Repeatability of both QC levels ranged from 3.96 to 8.32% for all the analytes. Intermediate precision ranged from 2.47 to 6.73% among all analytes for both QC levels. These data document the excellent precision of the method and the characterization of QCs to allow for evaluation of assay accuracy and precision for each analytical batch analyzed.

**TABLE 3:** Method precision, range, and linearity for VEA and other tocopherols.

Analyte	LOD ng/mL	Dynamic range ng/mL	Linearity (*R* ^2^; n = 7)	Precision (%RSD; n = 20)			
				Repeatability		Intermediate precision	
				QCL	QCH	QCL	QCH
VEA	1.53 (1.85 × 10^−5^)^a^	10–1,000 (1.21 × 10^−4^–0.0121)^a^	0.99	4.72	6.14	4.90	6.66
α-tocopherol	0.73 (8.85 × 10^−6^)^a^	10–1,000 (1.21 × 10^−4^–0.0121)^a^	0.99	4.36	4.96	6.73	6.00
δ-tocopherol	1.88 (2.28 × 10^−5^)^a^	10–1,000 (1.21 × 10^−4^–0.0121)^a^	0.99	8.32	6.06	2.98	4.56
γ- tocopherol	1.77 (2.15 × 10^−5^)^a^	10–1,000 (1.21 × 10^−4^–0.0121)^a^	0.99	4.86	3.96	2.47	4.16

a Units of µg analyte per mL aerosol puff volume.

The method demonstrates excellent linearity *R* ^2^ ≥ 0.99 for VEA and other tocopherols within the selected dynamic range of 10–1,000 ng/ml (Table 3). The sensitivity of the method is adequate to measure background levels of VEA (LOD = 1.85 × 10^−5^ μg per mL of aerosol puff volume (1.53 ng/ml methanol extract)) and other tocopherols (LODs: 8.85 × 10^−6^–2.28 × 10^−5^ μg per mL of aerosol puff volume (0.73–1.88 ng/ml methanol extract)) in aerosol emissions of EVPs liquid samples. The sensitivity for VEA detection was significantly better than previously published methods (LC-UV used to achieve an LOD of 580 ng/ml) (Brabcová et al., 2013). Our method was also 3–90 fold more sensitivity for α-tocopherol, δ-tocopherol, and γ-tocopherol compared with previously published methods (Bustamante-Rangel et al., 2007; Lanina et al., 2007; Cortés-Herrera et al., 2019).

### Method Application

The analytical method was applied to aerosol emissions from 147 EVPs associated with EVALI cases (Table 4). VEA and α-tocopherol had the highest detection rates of 46 and 41% respectively. VEA content in aerosol emissions ranged from 1.87 × 10^−4^to 74.1 µg per mL of aerosol puff volume followed by α-tocopherol with a range of 1.47 × 10^−2^ – 0.908 µg per mL of aerosol puff volume. VEA levels were 264 times higher than α-tocopherol with a mean of 25.1 µg per mL of aerosol puff volume compared to mean of 0.095 µg per mL of aerosol puff volume for α-tocopherol. Further quantification of VEA and α-tocopherol in e-liquid and in vaped aerosol will help provide insight about possible VEA degradation to form reactive byproducts such as ethenone (Wu and O’Shea, 2020). Gamma-tocopherol was detected in five EVPs while δ-tocopherol was not present in any of the analyzed products.

**TABLE 4:** VEA and other tocopherols concentrations and detection frequency in aerosol emissions of EVALI case-associated EVPs (µg per mL aerosol puff volume).

Analyte	N	% Detected	Mean ± Std Dev^a^
VEA	147	46	25.1 ± 22.4
α-tocopherol	126	41	0.095 ± 0.150
γ-tocopherol	112	5	0.0193 ± 0.0073
δ-tocopherol	112	0	NA

a Descriptive Statistics for detects only.

A subset of 139 products were stratified by active ingredient to further investigate the presence of macro-levels of VEA (>0.1%) in different product types. We evaluated macro-levels of VEA because VEA accumulation in the lungs could physically disrupt the tertiary structure of the alveolus, cause alveolar collapse, and subsequently lead to EVALI pathologies (Casals and Cañadas, 2012; Kamal and Raghunathan, 2012; Blount et al., 2020; Jonas and Raj, 2020). Products with higher VEA in aerosol emissions (>0.1% TPM) would deliver significant amounts of VEA to the lungs of people using the products. We show here that no nicotine or CBD products contain these high levels of VEA, and that 71% of case-associated THC products contained VEA as a macro-component (mean 32.0 µg per mL of aerosol puff volume). The high prevalence of VEA in THC products is consistent with the solubility of THC in VEA and the absence of VEA in nicotine products is consistent with the insolubility of nicotine in VEA. This result is also aligned with reported use of VEA as a diluent in the formulation of THC products (Downs, 2019a; Downs, 2019b; Zachary Eisenberg, 2019). VEA was also detected in two products with no THC, CBD, or nicotine with a mean level of 31.7 µg per mL of aerosol puff volume. One of these products was marketed as a THC-containing product by Dank Vapes but contained no detectable THC by our analysis. The high prevalence of macrolevel VEA in EVALI case-associated THC products further implicates VEA as a potential cause of vaping-associated lung injury (Cunha et al., 2006; Cilla et al., 2014).

## Conclusion

A rapid, isotope dilution LC-MS/MS method was developed for the simultaneous analysis of VEA and other tocopherols in EVP aerosol emissions. The method demonstrated high accuracy, precision, and sensitivity. VEA and other tocopherols, except for δ-tocopherol, were detected in aerosol emissions from EVALI case-associated EVPs; the mean VEA concentration was several orders of magnitude higher than the mean α-tocopherol concentration. VEA was predominantly found in THC products, consistent with the reported use of VEA as a diluent in the formulation of these products. Our results also indicate that VEA can be efficiently transferred to aerosol by EVALI-associated devices vaped using a standardized protocol. This method can serve as a valuable tool to improve surveillance for the potentially harmful additive VEA in EVPs.

## Data Availability

The raw data supporting the conclusions of this article will be made available by the authors, without undue reservation.

## Footnotes

1
https://www.cdc.gov/tobacco/basic_information/e-cigarettes

2
https://www.ncsl.org/research/health/state-medical-marijuana-laws.aspx

3
https://www.fda.gov/news-events/public-health-focus/lung-injuries-associated-use-vaping-products
